# Overexpression of Glycosyltransferase 8 Domain Containing 1 Promotes Gastric Cancer Proliferation and Inhibits Apoptosis via Mediating PTPN6/JAK2/STAT3 Signaling Axis

**DOI:** 10.7150/ijms.102719

**Published:** 2024-11-11

**Authors:** Yingying Sun, Wuqian Zhang, Qunyou Cong, Yanli Ge, Junjie Zhang, Haiyang Wang, Zhe Wang, Zhirong Wang

**Affiliations:** 1 Department of Gastroenterology, Tongji Hospital, School of Medicine, Tongji University, Shanghai, 200065, China.; 2 Department of Laboratory Medicine, Tongji Hospital, School of Medicine, Tongji University, Shanghai, 200065, China.

**Keywords:** gastric cancer, proliferation, apoptosis, GLT8D1, PTPN6, JAK2/STAT3

## Abstract

**Background:** The mechanisms of gastric cancer (GC) occurrence and development are still unclear. Although glycosyltransferase 8 domain containing 1 (GLT8D1) has been implicated in GC, its specific role and molecular mechanisms in GC progression need to be further investigated.

**Methods:** Tissue microarrays were used to detect the expression levels of GLT8D1 in 80 GC tissues and their corresponding non-tumor adjacent tissues. The correlations between the GLT8D1 expression level and clinicopathological characteristics were evaluated. A series of *in vitro* and *in vivo* functional experiments were performed to explore the role of GLT8D1 in GC progression. Combined with transcriptomic RNA sequencing (RNA-seq) and Weighted Gene Co-expression Network Analysis (WGCNA), we delineated the potential mechanisms via experimental verification.

**Results:** Elevated expression of GLT8D1 in GC tissues was positively correlated with advanced clinical stages and poor prognosis. Konckdown of GLT8D1 significantly inhibited GC cell proliferation and induced apoptosis, whereas overexpression did the opposite. Further researches demonstrated that protein tyrosine phosphatase non-receptor type 6 (PTPN6), a downstream target of GLT8D1, has the capacity to modulate the activity of the JAK2/STAT3 signaling pathway.

**Conclusions:** Our study indicated that GLT8D1 expression was upregulated in GC tissues and correlated with poor prognosis. We reveal a potential molecular mechanism by which GLT8D1 promotes GC progression.

## Introduction

Gastric cancer (GC) is one of the most prevalent malignant tumors in the world and ranks fifth among the most common cancers globally^1^, ^2^. Its evasive early symptoms and low screening rates often lead to late diagnoses, making GC the fourth leading cause of cancer-related deaths^1^-^3^. And despite the declining trend in morbidity and mortality over the past century, GC continues to represent a considerable socioeconomic burden^1^. Established risk factors for GC include* Helicobacter pylori* infection, *Epstein-Barr virus* infection, smoking, obesity, and the excessive consumption of salt and processed meats. These factors are well-studied and acknowledged as significant contributors to GC development. Addressing these modifiable risk factors through public health initiatives and clinical interventions may help reduce the incidence of this malignancy^2^. The diagnosis of GC typically relies on a combination of clinical symptoms, endoscopic examination, imaging studies, serological tests, and pathological assessments^4^-^6^. With the advancement of multi-omics analysis techniques, a multitude of biomarkers associated with the diagnosis and prognosis of GC have been identified. Further exploration of the biological processes and pathways reflected by these markers is essential to fully comprehend their role and effectively apply this knowledge to clinical practice.

Glycosylation is a critical post-translational modification of proteins, and recent studies have shown that aberrant glycosylation is associated with cancer occurrence and progression as well as drug resistance and other malignant phenotypes^7^, ^8^. Abnormal glycosylation may arise from altered expression or specific activity changes of glycosyltransferases and glycosidases. In the last few years, attention has been drawn to the link between dysregulated expression of glycosyltransferases and cancer development^9^-^13^. One enzyme of interest, Glycosyltransferase 8 domain containing 1 (GLT8D1), has been associated with the progression of various cancers, such as head and neck squamous cell carcinoma, melanoma, and glioma^14^-^18^. Previous research has indicated by bioinformatics analysis that GLT8D1 may serve as a potential prognostic marker for poor prognosis in GC and could be associated with tumor immunity^19^. However, the underlying mechanisms by which GLT8D1 impacts GC are not fully understood.

Our research demonstrates that GLT8D1 is upregulated in GC tissues, and higher expression levels indicate more advanced tumor stage and poorer prognosis, suggesting that it has the potential to be a novel biomarker for GC progression. *In vitro* functional assays reveal that GLT8D1 knockdown effectively inhibits the proliferation of GC cells, induces apoptosis, and leads to G1 phase cell cycle arrest. Conversely, overexpression of GLT8D1 produces opposite results. Subsequently, we further validated these findings *in vivo*. Moreover, our study uncovers a significant link between GLT8D1 and protein tyrosine phosphatase non-receptor type 6 (PTPN6). GLT8D1 affects the activation state of the JAK2/STAT3 signaling pathway by influencing the expression of PTPN6. This discovery highlights the pivotal role of GLT8D1 in the progression of GC, suggesting that it may be recognized as a novel biomarker.

## Materials and methods

### Bioinformatics analysis

Gene expression datasets and associated clinical profiles of GC patients were obtained from The Cancer Genome Atlas (TCGA, https://portal.gdc.cancer.gov/) database. The gene set specific to glycosyltransferase was sourced from the Gene Ontology (GO, http://geneontology.org/) database. The downloaded datasets underwent a standardized preprocessing protocol to ensure data integrity and comparability. This was followed by comprehensive subsequent analyses using various R packages.

### Patients and information collection

GC tissues and matched corresponding adjacent non-cancerous tissues were collected from a cohort of 80 patients who underwent surgical resection at Shanghai Tongji Hospital. The collection of tissues was performed with the patients' informed consent, ensuring the study's conduct in accordance with Declaration of Helsinki. The clinical and pathological information of each patient were acquired from the medical records of Shanghai Tongji Hospital. The extraction of data was used solely for research purposes. All procedures were conducted in compliance with standard procedures approved by the Medical Ethics Committee of Shanghai Tongji Hospital.

### Cell culture and lentiviral transduction

The GC cell lines and the normal gastric epithelial cell line GES-1 were procured from the Cell Bank of the Chinese Academy of Sciences. MKN28, HGC-27, SGC-7901 and GES-1 cells were cultured in RPMI-1640 medium (Servicebio) supplemented with 10% Fetal Bovine Serum (FBS, FuHeng Biology). The AGS cell line was cultured in F-12K medium (Servicebio) containing 10% FBS (FuHeng Biology). All cell lines were incubated in a humidified incubator at 37 °C and 5% CO2.

The human GLT8D1 complementary DNA (cDNA) was amplified via Polymerase Chain Reaction (PCR) and subsequently cloned into the pLV3-CMV-3xFLAG-Puro lentivirus vector, with successful integration confirmed through sequencing. Short hairpin RNA (shRNA) sequences targeting GLT8D1 were incorporated into the pLV3-U6-Puro lentiviral vector for knockdown experiments. For lentiviral particle production, both overexpression and knockdown constructs were co-transfected with packaging plasmids pSPAX2 and pMD2.G into 293T cells, following the manufacturer's protocol. The cell culture supernatants containing lentiviral particles were harvested 48 hours post-transfection. The cells were then transduced with the collected lentivirus, and stable integrants were selected using 2 µg/mL puromycin (Beyotime). Sequences of shRNAs are available in Supplementary Table S1.

### Quantitative Reverse Transcription-Polymerase Chain Reaction (qRT-PCR)

Total RNA was extracted using TRIzol (Invitrogen). 1ug total RNA was reverse transcribed to cDNA with PrimeScript RT Reagent Kit (Takara) according to the manufacturers' instruction. qPCR was performed using SYBR Green 2xTaq mix (Takara) and analyzed with QuantStudio 6 Operating Software. Glyceraldehyde-3-phosphate dehydrogenase (GAPDH) was employed as an endogenous control to normalize the expression levels. The relative expression levels of the target genes were quantified using the comparative Ct (2^-ΔΔCt^) method. The primer sequences are listed in Supplementary Table S1.

### Western blot

Total protein was extracted by utilizing Radio-Immunoprecipitation Assay (NCM Biotech) buffer supplemented with protease and phosphatase inhibitors cocktail (APExBIO) and was qualified by Bicinchoninic Acid (Beyotime) reagent kit. Equal quantities of total protein were separated by Sodium Dodecyl Sulfate-Polyacrylamide Gel Electrophoresis (Beyotime) and transferred onto Polyvinylidene Fluoride (Immobilon) membrane, and blocked with 5% skim milk powder for 1 hour at room temperature. After incubation with the primary antibody at 4 °C overnight, the membrane was washed with Tris-Buffered Saline with Tween (TBST, Servicebio) buffer three times and incubated with secondary antibodies included horseradish peroxidase (HRP) -goat anti-mouse (Beyotime), HRP-goat anti-rabbit (Beyotime) 1 hour at room temperature. Finally, signal on the membrane was visualized by Enhanced Chemiluminescence system. A list of antibodies is provided in Supplementary Table S2.

### Hematoxylin and eosin (H&E) staining

Tissue sections were roasted at 60 °C firstly. After paraffin sections were dewaxed and hydrated, the nucleus was stained with hematoxylin (Solarbio), and then cytoplasmic staining was carried out with eosin staining solution (Solarbio). After the slides were dried, the sheets were preserved with a neutral resin (Biosharp).

### Immunohistochemistry (IHC) staining

The slides were incubated at 60 °C until the paraffin melts, followed by dewaxed in xylene and rehydrated with gradient ethanol concentrations solutions. The tissue sections were then subjected to antigen repair, followed by blocking of the inactivated endogenous peroxidase with 3% hydrogen peroxide solution. Subsequently, blocked sections were incubated with indicated primary antibody overnight at 4 °C in a wet box. After washing three times with Phosphate-Buffered Saline with Tween-20 (PBST, Servicebio), the slides were incubated with the indicated HRP conjugated secondary antibody at room temperature for 1 hour and washed with PBST. Then the tissue sections were stained with diaminobenzidine (Vector), and the sections were counterstained with hematoxylin (Solarbio). Immunoreactive scores were calculated by multiplying the scores of staining signal intensity and the percentage of positive cells. Tissues with scores ≤ 6 were defined as low expression, and those with scores > 6 were defined as high expression. A list of antibodies is provided in Supplementary Table S2.

### Cell Counting Kit-8 (CCK-8) assay

CCK-8 assay was applied to assess the cell proliferation according to the manufacturer protocol. A total of 2000 cells in 100ul cell suspension were plated into each well of a 96-well plate and culture for indicated times. Then, 10ul CCK-8 reagent (NCM Biotech) was added into each well incubated for 2 hours at 37°C. The absorbance was measured by a microplate spectrophotometer at 450 nm.

### Colony formation assay

For the colony formation assay, a total of 400 cells were seeded in each well of a 6-well plate and cultured for 14 days. Then the colonies of cells were fixed with 4% paraformaldehyde (Beyotime), stained with 0.1% crystal violet (Beyotime). The exact colony number of colonies was then quantified.

### 5-Ethynyl-2'-deoxyuridine (EdU) assay

A total of 6000 indicated cells were plated in each well of 24-well plates for EdU assay. According to the EdU manufacturer protocol, 2 hours before fixation, the cells were pre-treated with 10 μM EdU (Epizyme) at 37 ˚C, followed by fixation with 4% paraformaldehyde (Beyotime) and then permeabilized with 0.3% Triton X-100 (Beyotime). After completing the above steps, 100ul click additive solution was added into each well for 30 mins at room temperature and keep in dark place. 4',6-diamidino-2-phenylindole (DAPI) was used to stain the cell nuclei. Samples were imaged by a Nikon Ti fluorescence microscope and quantified.

### Immunofluorescence

Cells were fixed with 4% paraformaldehyde (Beyotime) for 20 mins at room temperature. Following 1 hour blocking in 10% goat serum at room temperature, the cells were incubated with the indicated primary antibody overnight at 4 °C. After that, the cells were incubated with the corresponding fluorescent secondary antibodies and DAPI. The samples were visualized with inverted fluorescence microscope. A list of antibodies is provided in Supplementary Table S2.

### Flow cytometry analysis

For the cellular apoptosis assay, indicated cells were harvested and fixed with 70% ethanol at 4 °C overnight, then stained with Annexin V-FITC (Yeasen) and propidium iodide (PI, Yeasen) and subsequently the ratio of apoptotic cells was tested by flow cytometry. For cell cycle analysis, the fixed cells were stained with PI and then examined by flow cytometry. A total of 30,000 events were recorded for each sample and analyzed by FlowJo and Modfit software.

### Tumor xenograft

Four-week-old female BALB/c nude mice were purchased from Vital River Laboratory (Beijing, China). The mice were randomly divided into indicated groups and injected with HGC-27 cells stably infected with lentivirus subcutaneously. The tumor lengths (L) and widths (W) were measured using a caliper every 4 days, and the volume (V) was calculated with the formula: V = (L × W^2^)/2. Four weeks later, all the mice were euthanized and the tumors were harvested. All animal experiments were approved by the Animal Care and Use Committee at Shanghai Tongji Hospital.

### Statistical analysis

The statistical analysis was conducted using GraphPad Prism software and R software. The association between GLT8D1 and the clinicopathologic parameters of the GC patients were evaluated by a Chi-square test. The Kaplan-Meier method was utilized to evaluate the correlation between GLT8D1 expression and GC patient survival. The Student's t-test was used to determine the statistical significance of differences between groups. Data were presented as mean ± SD. P<0.05 was considered statistically significant.

## Results

### Expression and prognosis analysis of GLT8D1 in the database

**Figure 1:**
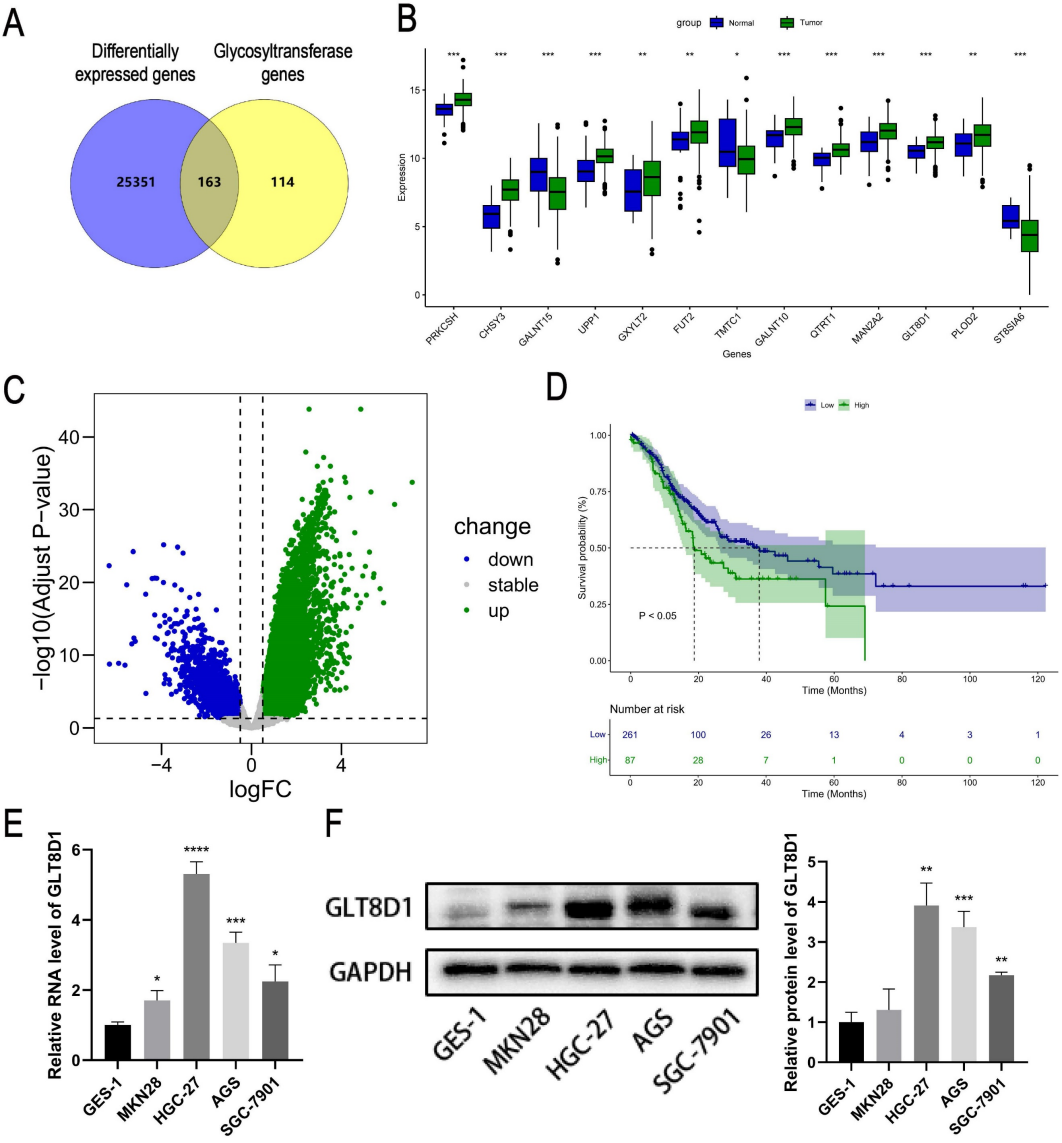
** Expression and prognosis analysis of GLT8D1 in the database. (A)** The intersection genes between DEGs related to GC in the TCGA database and the glycosyltransferase genes by Venny diagram. **(B)** Expression profiles of GC prognostic-associated genes. **(C)** Volcano plot of DEGs related to GC in the TCGA. **(D)** Kaplan-Meier survival curve analysis of GLT8D1 in GC from TCGA. **(E)** The relative expression of GLT8D1 in GC and GES-1 cell lines by qRT-PCR. **(F)** Protein expression of GLT8D1 in GC and GES-1 cell lines by Western blot. The data are presented as the mean ± SD, *p < 0.05, **p < 0.01, ***p < 0.001, ****p < 0.0001.

Initially, we intersected the differentially expressed genes (DEGs) related to GC in the TCGA database with genes from glycosyltransferase database, a total of 163 genes with potential associations were identified (Figure 1A). Subsequently, the Cox regression analysis revealed that 13 of these genes were significantly correlated with the prognosis of patients with GC (Figure 1B, Table 1). According to the TCGA database, GLT8D1 expression was elevated in GC, and that individuals with high levels of GLT8D1 had a significantly poorer prognosis compared to those with low levels (Figure 1C, D). To validate these findings, we examined the endogenous expression of GLT8D1 in a panel of GC cell lines, including MKN28, HGC-27, AGS, SGC-7901, and the human normal gastric epithelial cell line GES-1 using qRT-PCR and Western blot. Our results demonstrated that GLT8D1 was overexpressed in GC cells compared to the normal gastric epithelial cells at both RNA and protein levels (Figure 1E, F). Based on these findings, we selected the HGC-27 and AGS cell lines for subsequent experiments.

### GLT8D1 is highly expressed in GC and correlates with poor prognosis

**Figure 2:**
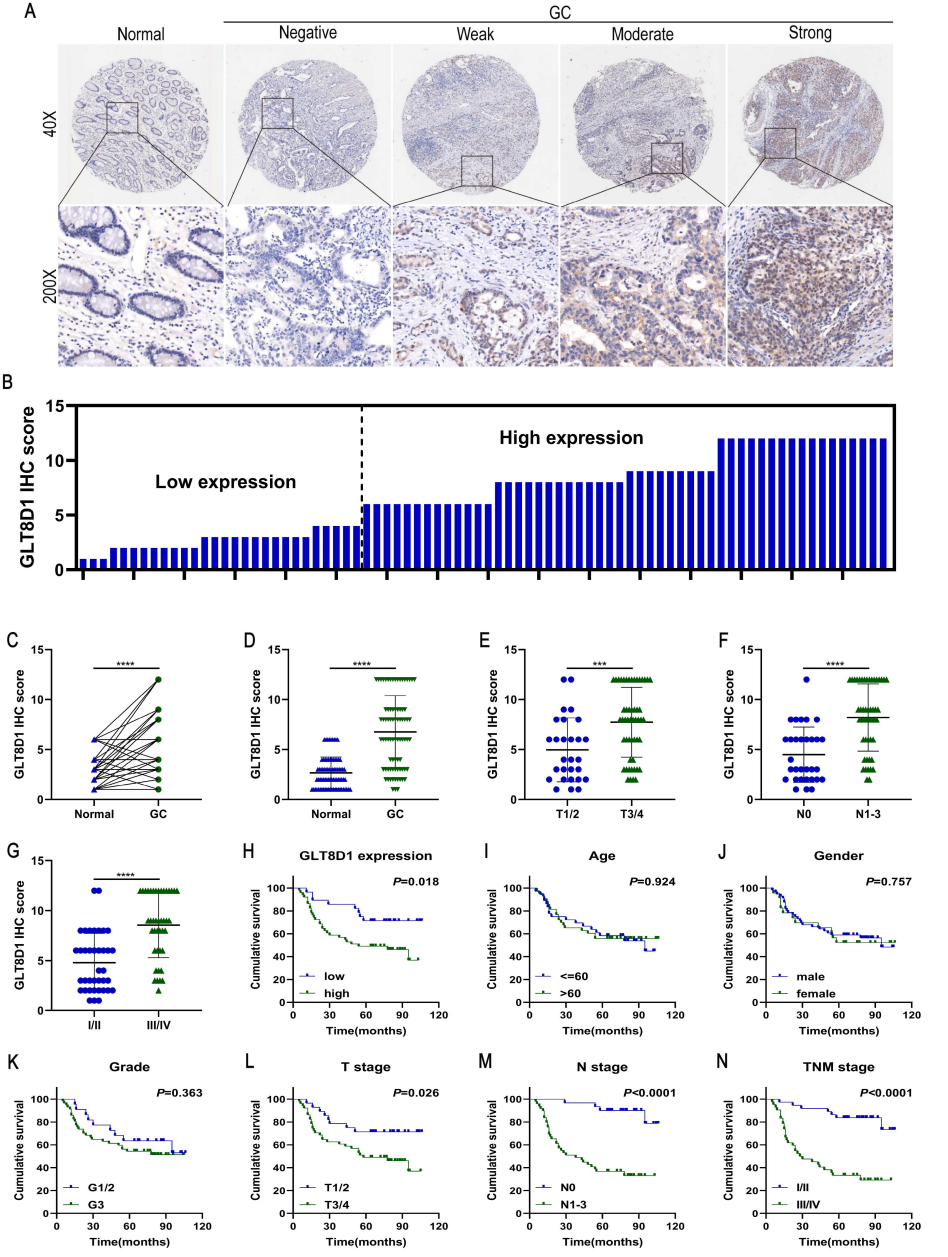
** Overexpression of GLT8D1 is observed in GC and correlates with poor prognosis. (A)** Representative images showed the expression of GLT8D1 in 80 pairs of GC and the adjacent normal tissues detected by IHC. **(B)** The IHC scores were calculated in 80 GC tissues. **(C, D)** GLT8D1 expression was quantified in 80 pairs of GC and the adjacent normal tissues.** (E-G)** Dot distribution graph of GLT8D1 IHC scores was shown in 80 GC patients of different clinical stages. **(H-N)** Kaplan-Meier survival curve analysis indicated that the OS of GC patients with high expression of GLT8D1 was significantly shorter than that of GC patients with low expression of GLT8D1. The OS was significantly correlated with T stage, N stage and TNM stage, but not with age, gender and grade. The data are presented as the mean ± SD, *p < 0.05, **p < 0.01, ***p < 0.001, ****p < 0.0001.

To analyze the expression and clinical value of GLT8D1, IHC was conducted to determine GLT8D1 protein levels in 80 pairs of GC tissues and their corresponding adjacent non-cancerous tissues (Figure 2A, B). The findings revealed that GLT8D1 was significantly upregulated in GC tissues compared with the adjacent normal tissues (Figure 2C, D). Subsequently, we evaluated the correlation between GLT8D1 expression and clinicopathologic characteristics (Table 2). Our results indicated elevated GLT8D1 was positively correlated with advanced T stage (p = 0.011), N stage (p = 0.003), and TNM stage (p = 0.002), while no significant correlations were observed with other clinical features such as age, gender, and grade. Further analysis confirmed a positive association between the GLT8D1 IHC score and the T stage, N stage and TNM stage (Figure 2E-G). We then analyzed the influence of GLT8D1 expression on patient overall survival (OS). Kaplan-Meier survival curves demonstrated that patients with high GLT8D1 expression had a significantly worse prognosis (Figure 2H). In the present study, age, gender and grade appeared to have no effect on the OS of patients (Figure 2I-K). Moreover, the Kaplan-Meier curves illustrated that patients with T3/4 stage (p=0.026), N1-3 stage (p<0.0001) and TNM stage III/IV (p<0.0001) exhibited shorter OS compared to those with early stages (Figure 2L-N). Overall, our study establishes that GLT8D1 is overexpressed in GC and significantly associated with a poor prognosis, highlighting its potential as a biomarker for GC progression.

### GLT8D1 promotes GC cell proliferation

**Figure 3:**
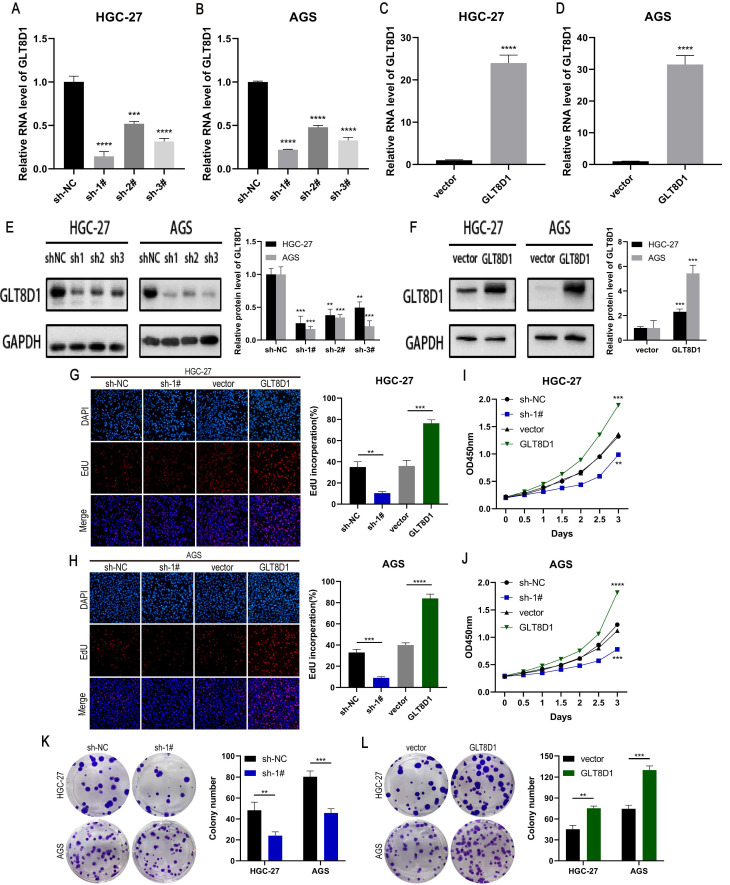
** GLT8D1 promotes GC cell proliferation. (A-D)** Relative expression of GLT8D1 was measured by qRT-PCR in GLT8D1 knockdown and overexpression GC stable cell lines.** (E, F)** The protein level of GLT8D1 was detected by western blot in stable cell lines.** (G, H)** EdU assay was performed in GLT8D1 stable cell lines.** (I, J)** The growth curves of cells were evaluated by using CCK-8 assay. **(K, L)** Colony formation assay was performed to evaluate cell proliferation. The data are presented as the mean ± SD, *p < 0.05, **p < 0.01, ***p < 0.001, ****p < 0.0001.

To verify the role of GLT8D1 in GC, we established stable cell lines with GLT8D1 knockdown or overexpression in HGC-27 and AGS cells using shRNA and lentiviral vectors. QRT-PCR and Western blot analysis confirmed significant downregulation or upregulation of GLT8D1 expression, respectively (Figure 3A-F). Among the three shRNAs, sh1# exhibited the highest silencing efficiency and was selected for subsequent experiments. EdU assay, CCK8 assay and colony formation assay were used to evaluated the effect of GLT8D1 on GC cell proliferation. The EdU assay showed that GLT8D1 knockdown significantly reduced the proliferation capacity of GC cells, while its overexpression significantly enhanced cell proliferation relative to controls (Figure 3G, H). Consistent with the EdU assay, both CCK8 assay and colony formation assay indicated that GLT8D1 promotes the growth of GC cells (Figure 3I-L). Taken together, these findings establish GLT8D1 as a crucial regulator of GC cell proliferation.

### GLT8D1 deficiency promotes GC cell apoptosis

**Figure 4:**
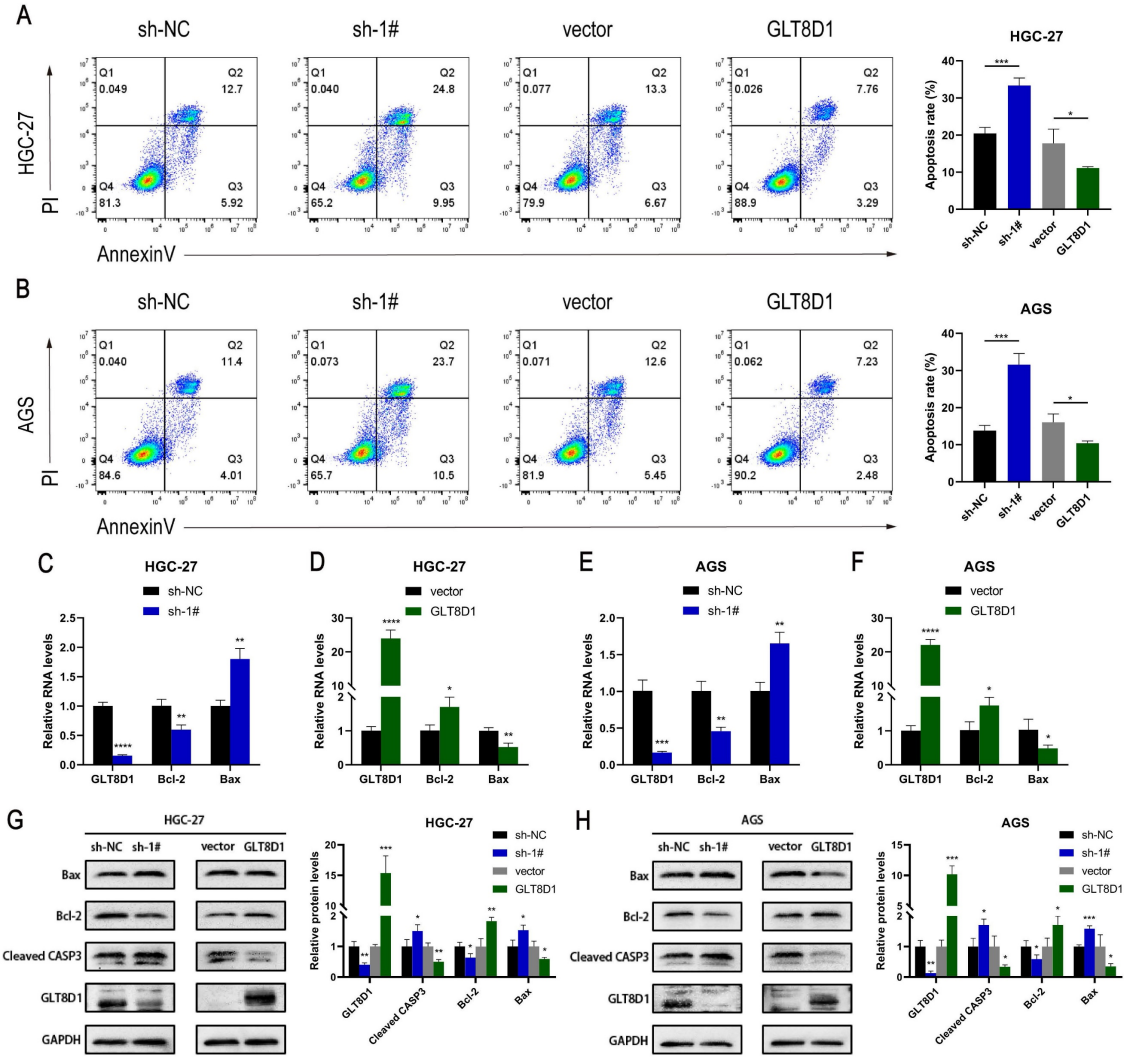
** GLT8D1 depletion promotes GC cell apoptosis. (A, B)** Apoptosis rate was performed using flow cytometry in GC cell lines. **(C-F)** Apoptosis-related RNA levels were assessed by qRT-PCR. **(G, H)** Apoptosis-related protein levels were monitored by western blot. The data are presented as the mean ± SD, *p < 0.05, **p < 0.01, ***p < 0.001, ****p < 0.0001.

To elucidate the influence of GLT8D1 on apoptosis of GC cells, we conducted flow cytometry analysis following Annexin V/PI double staining to assess the apoptotic rates among cells with modulated GLT8D1 expression. The analysis revealed that GC cells with GLT8D1 knockdown exhibited significantly higher apoptosis rates compared to the control group (Figure 4A, B). In contrast, the overexpression of GLT8D1 was associated with a reduction in apoptotic rates relative to the control group. QRT-PCR indicated that GLT8D1 suppression resulted in a significant upregulation of pro-apoptotic genes and a downregulation of anti-apoptotic genes (Figure 4C-F). Conversely, the overexpression of GLT8D1 produced the opposite effects on the expression of these genes. Furthermore, Western blot was used to examine the expression of apoptosis-related proteins. The results demonstrated that the levels of the pro-apoptotic proteins Bax and cleaved caspase-3 were markedly increased in the GLT8D1 knockdown group, while the anti-apoptotic protein Bcl-2 was significantly reduced. The overexpression of GLT8D1 induced the inverse changes in protein expression (Figure 4G, H). These findings collectively indicate that GLT8D1 depletion promotes apoptosis in GC cells.

### GLT8D1 expression regulates the cell cycle

**Figure 5:**
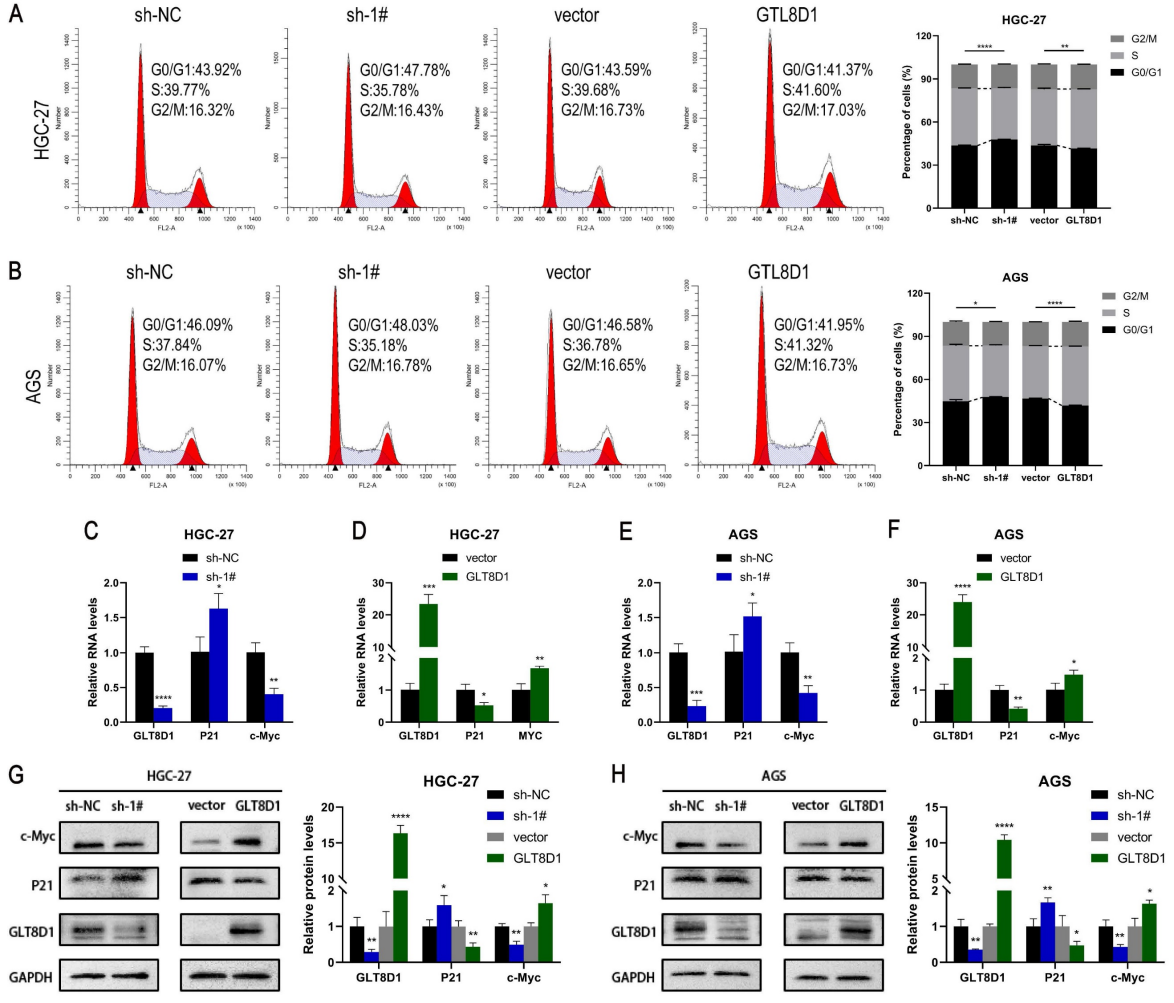
** GLT8D1 expression regulates the cell cycle. (A, B)** Flow cytometry was utilized to assess the influence of GLT8D1 on cell cycle progression. **(C-F)** qRT-PCR was conducted to determine the RNA levels of the cell cycle-related genes. **(G, H)** Cell cycle-related protein levels were measured by western blot. The data are presented as the mean ± SD, *p < 0.05, **p < 0.01, ***p < 0.001, ****p < 0.0001.

The cell cycle analysis examined by flow cytometry demonstrated that knockdown of GLT8D1 resulted in an increase in G1 phase GC cells and a decrease in S phase GC cells, suggesting G1 phase block after knockdown of GLT8D1 (Figure 5A, B). In contrast, overexpression of GLT8D1 was associated with accelerated cell cycle progression. Subsequently, qRT-PCR analysis indicated that downregulation of GLT8D1 led to diminished RNA levels of the cell cycle-promoting gene c-Myc and increased levels of the cell cycle-inhibiting gene P21. Overexpression of GLT8D1, on the other hand, led to an inverse effect (Figure 5C-F). Moreover, Western blot assay substantiated that the induction of G1 cell cycle arrest by GLT8D1 depletion was likely due to elevated P21 expression and reduced c-Myc expression (Figure 5G, H).

### Down-regulation of GLT8D1 inhibits the growth of xenograft tumors *in vivo*

**Figure 6:**
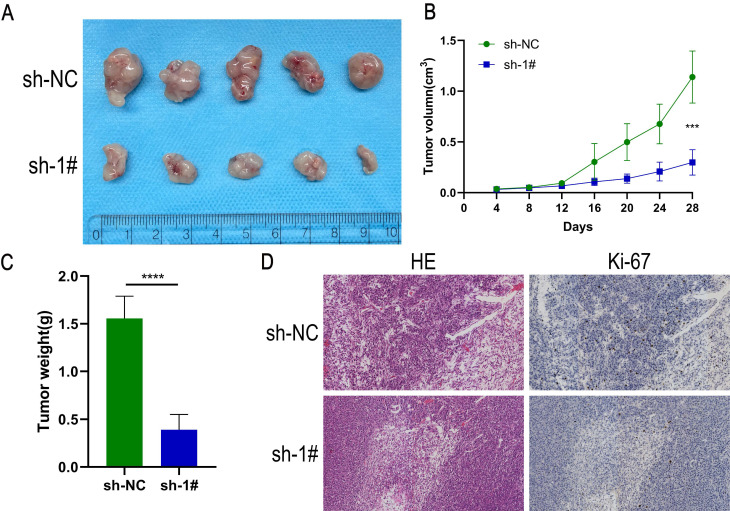
** Down-regulation of GLT8D1 inhibits the growth of xenograft tumors *in vivo*. (A)** Tumor growth of mice implanted subcutaneously with HGC-27 cells. **(B)** Tumor volumes were measured every 4 days. **(C)** Final tumor weight was analyzed. **(D)** Sections of subcutaneously implanted tumors were stained with H&E and IHC, showing representative images. The data are presented as the mean ± SD, *p < 0.05, **p < 0.01, ***p < 0.001, ****p < 0.0001.

To evaluate the impact of GLT8D1 on tumor growth *in vivo*, a xenograft tumor model was established by injecting HGC-27 cells with GLT8D1-stabilized knockdown and control cells into female nude mice (Figure 6A). The tumor volume and weight were significantly reduced in the GLT8D1 knockdown group compared with the control group (Figure 6B, C). Furthermore, IHC provided evidence that the downregulation of GLT8D1 significantly suppresses tumor growth, as indicated by a marked decrease in the expression of the proliferation marker Ki-67 in the tumors with GLT8D1 knockdown (Figure 6D). These results were consistent with assays *in vitro*, suggesting that GLT8D1 could promote GC progression.

### GLT8D1 exerts an influence on the expression levels of PTPN6

**Figure 7:**
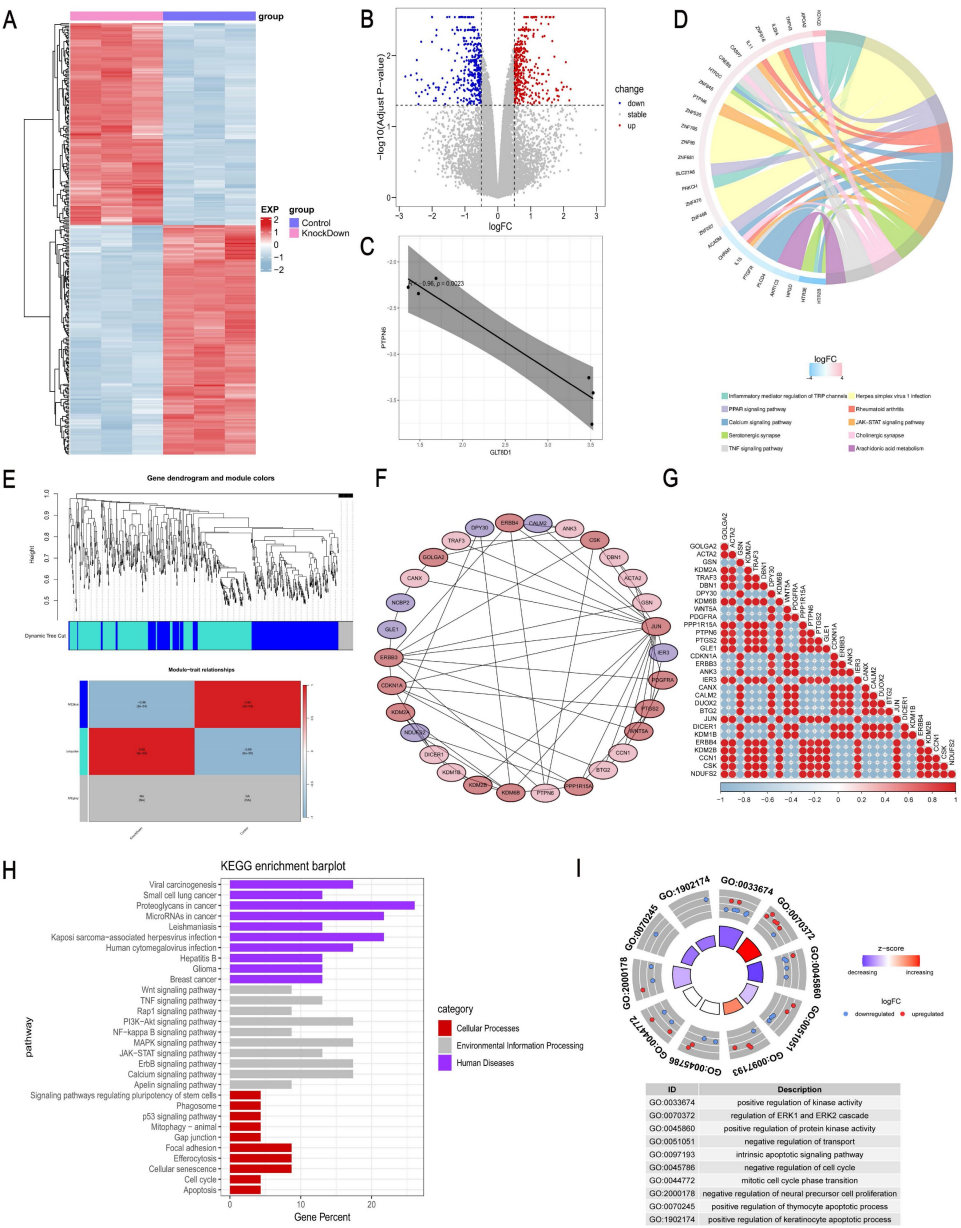
** GLT8D1 exerts an influence on the expression levels of PTPN6. (A)** Heatmap of DEGs between GLT8D1 stable knockdown cells and the negative control cells identified from RNA-seq. **(B)** Volcano plot of RNA-seq data. **(C)** The correlation analysis between PTPN6 and GLT8D1. **(D)** The KEGG enrichment analysis of DEGs.** (E)** WGCNA on the DEGs. **(F)** Construction of PPI network to identify hub genes.** (G)** The correlation analysis of hub genes. **(H)** The KEGG enrichment analysis of hub genes.** (I)** The GO enrichment analysis of the hub gene set.

To further determine the functional mechanism of GLT8D1 in GC cells, we performed transcriptomic RNA sequencing (RNA-seq) of HGC-27 cells with stable knockdown of GLT8D1 and the corresponding negative control cells, with three repetitions in each group (Figure 7A). Based on the sequencing results, a total of 244 DEGs were identified, of which 118 were up-regulated while 126 were down-regulated (fold change >2, p < 0.05; Figure 7B). A significant negative correlation was observed between PTPN6 and GLT8D1 expression levels (Figure 7C). Further KEGG enrichment analysis indicated that PTPN6 was significantly enriched in the JAK/STAT signaling pathway, with a top ten ranking of enrichment significance (Figure 7D). Subsequently, we further conducted a Weighted Gene Co-expression Network Analysis (WGCNA) on the DEGs to explore the relationships between gene co-expression modules and phenotypic traits (Figure 7E). Furthermore, we constructed a PPI network to identify 30 hub genes that are central to the biological processes and signaling pathways (Figure 7F, G). As anticipated, PTPN6 emerged as a potential target in our research. KEGG and GO enrichment analyses on the hub gene set revealed significant enrichment of biological processes and signaling pathways related to cell growth and apoptosis (Figure 7H, I). Notably, the JAK/STAT signaling pathway was significantly enriched and stood out in this context, underscoring its potential role in mediating the effects of GLT8D1.

### PTPN6/JAK2/STAT3 pathway was responsible for downstream regulation of GLT8D1

**Figure 8:**
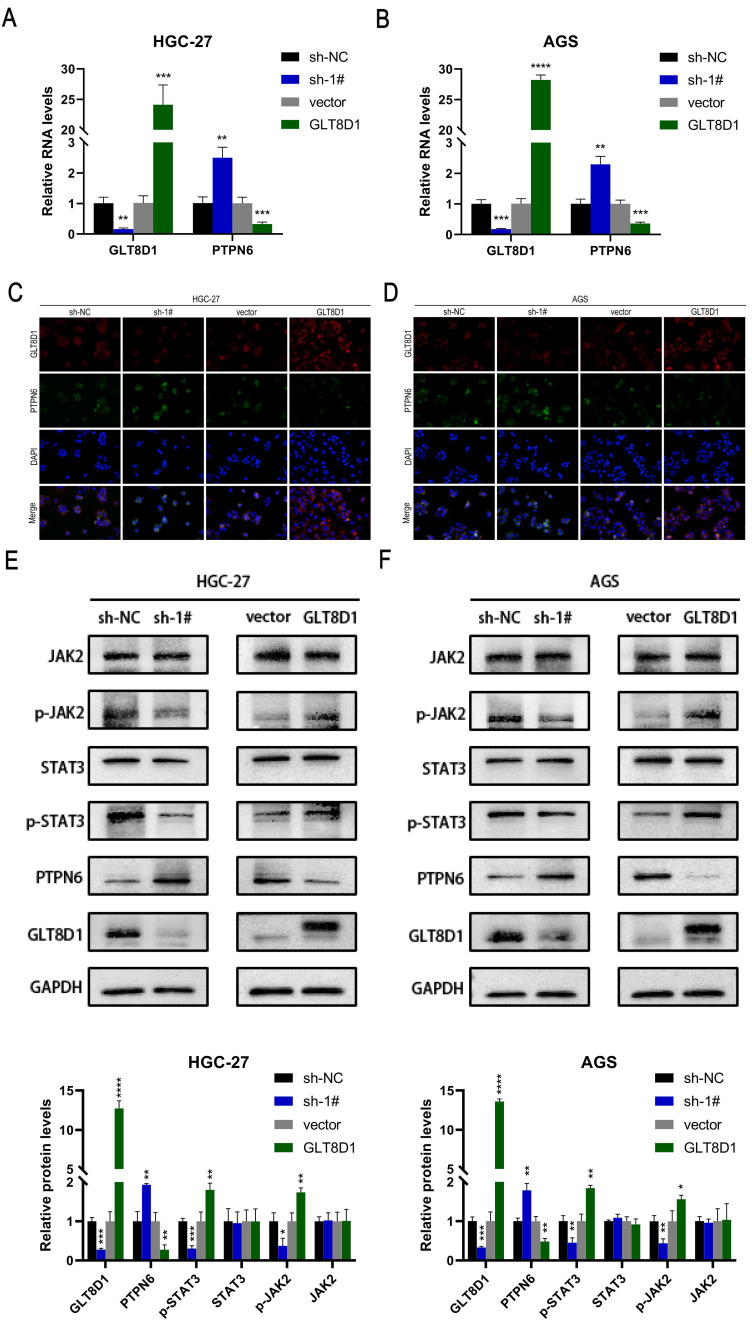
** PTPN6/JAK2/STAT3 axis was responsible for downstream regulation of GLT8D1. (A, B)** qRT-PCR verify the impact of GLT8D1 on the RNA level of PTPN6. **(C, D)** Representative images of Immunofluorescence staining.** (E, F)** Western blot analysis indicated the differential levels of JAK2/STAT3-related proteins after knockdown or overexpressing GLT8D1 in GC cell lines. The data are presented as the mean ± SD, *p < 0.05, **p < 0.01, ***p < 0.001, ****p < 0.0001.

To ascertain the impact of GLT8D1 expression on PTPN6 levels, we conducted a comprehensive set of experiments. QRT-PCR results revealed that PTPN6 RNA levels were significantly increased upon GLT8D1 suppression and decreased with GLT8D1 overexpression in HGC-27 and AGS cell lines (Figure 8A, B). Western blot analysis confirmed this trend, and PTPN6 protein levels were adjusted accordingly (Figure 8E, F). Immunofluorescence experiments further supported these findings, showing PTPN6 co-localized with GLT8D1 and suggesting that GLT8D1 negatively regulates PTPN6 expression (Figure 8C, D). Subsequently, we investigated the influence of altered GLT8D1 expression on the activity of the JAK2/STAT3 signaling pathway. Our findings showed that the suppression of GLT8D1 expression enhanced PTPN6 levels, which in turn results in the inactivation of the JAK2/STAT3 signaling pathway (Figure 8E, F). Overexpression of GLT8D1, however, results in the opposite effect. Consequently, our results demonstrate that PTPN6 serves as a downstream target of GLT8D1, with the capacity to modulate the activity of the JAK2/STAT3 signaling pathway.

## Discussion

GC is one of the most common malignant tumors and represent a substantial threat to global health^1^-^3^. The etiology of GC is intricately complex, and has not been fully elucidated for a comprehensive understanding^20^-^22^. Abnormal glycosylation has emerged as a critical factor in tumorigenesis and cancer progression, attracting increasing attention from the scientific community^7^, ^8^. By intersecting DEGs associated with GC from the TCGA database with those in glycosyltransferase database, we identified 163 putative GC-related glycosyltransferase genes. Subsequently, through Cox regression analysis, we found that GLT8D1 expression was upregulated in GC and was strongly associated with patient prognosis. Previous studies have reported that GLT8D1 is upregulated in multiple malignant tumors, such as head and neck malignancies, melanoma, and glioma, and is significantly correlated with prognosis^14^-^18^. Its deeper molecular mechanisms affecting the progression of GC remain largely unknown^19^. The present study delineates a novel regulatory role for GLT8D1, which modulates the JAK2/STAT3 signaling pathway by influencing PTPN6 levels, with consequential effects on GC cells proliferation and apoptosis.

To confirm the increased expression of GLT8D1 in GC, we quantified GLT8D1 levels in GES-1 cell line and a panel of GC cell lines. As anticipated, GLT8D1 was markedly upregulated in GC cells at both the transcriptional and translational levels. Extending our investigation, we analyzed GLT8D1 protein levels in a cohort of 80 GC tissues and adjacent non-cancerous tissues. Our data consistently showed that GLT8D1 was upregulated in GC tissues, and its elevated levels were associated with a more severe prognosis. Notably, elevated GLT8D1 expression was significantly correlated with advanced clinical pathological parameters, including T stage, N stage, and TNM stage. Our results provide compelling evidence that overexpression of GLT8D1 is associated with GC, suggesting that it may serve as a prognostic indicator for patient outcomes. To explore the biological functions of GLT8D1 in GC progression, lentiviruses were utilized to knockdown or overexpress GLT8D1 in HGC-27 and AGS GC cell lines^23^, ^24^. Subsequently, a series of *in vitro* and vivo experiments were conducted to verify the malignant features of GLT8D1 in GC. The results demonstrated that GLT8D1 knockdown significantly curtailed GC cell proliferation, stimulated apoptosis, and induced cell cycle arrest at the G1 phase. In contrast, GLT8D1 overexpression resulted in opposing phenotypes. Strikingly, GLT8D1 knockdown also markedly suppressed tumor growth in a xenograft mouse model, underscoring its pivotal role in GC progression.

In this study, we investigated the expression profiles of GLT8D1-knockdown GC cells in comparison to control GC cells using RNA-seq. Our KEGG enrichment analysis particularly highlighted the JAK2/STAT3 signaling pathway, which has been demonstrated in the regulation of cell proliferation, apoptosis, and other hallmarks of malignancy^25^-^28^. To further elucidate the underlying molecular mechanisms, we conducted WGCNA on the DEGs associated with GLT8D1. By constructing a PPI network, we identified a set of hub genes that are pivotal to biological processes and the signaling pathways. Notably, PTPN6, one of the hub genes, is likely to be a potential downstream regulator of GLT8D1. Previous researches have reported that PTPN6 can mediate the inhibition of the JAK2/STAT3 signaling pathway^29^-^32^. Based on these findings, we hypothesized that GLT8D1 modulates the JAK2/STAT3 signaling pathway by regulating the expression levels of PTPN6, which in turn affects the proliferation and apoptosis of GC cells.

Our findings clearly demonstrate that the suppression of GLT8D1 can significantly inhibit the activity of the JAK2/STAT3 signaling pathway. Our analysis encompassed not only proteins in the active forms of the pathway, including phosphorylated JAK2 (p-JAK2) and phosphorylated STAT3 (p-STAT3), but also a spectrum of downstream targets. These targets include anti-apoptotic proteins such as Bcl-2 and proliferation-related proteins such as P21 and c-Myc^28^, ^33^-^35^. This indicates that GLT8D1 indeed affects the activity of the JAK2/STAT3 signaling pathway, thereby regulating the proliferation and apoptosis of GC cells, which is consistent with previous research^36^. PTPN6 is a cytoplasmic tyrosine phosphatase that negatively regulate a variety of signaling pathways and is considered a tumor suppressor gene^37^-^39^. In our investigation, we revealed a negative correlation between GLT8D1 and PTPN6 expression levels. Specifically, GLT8D1 knockdown was associated with increased PTPN6 expression, while GLT8D1 overexpression induced the opposite effect. It has been shown that the antitumor effects of PTPN6 are primarily achieved by directly regulation of JAK2 and STAT3, thereby exerting a negative control on the oncogenic signaling pathway of STAT3^38^, ^40^-^43^. Based on these findings, our study identified PTPN6 as a downstream target of GLT8D1, which is essential for various cellular processes.

Our study presents compelling evidence that GLT8D1 is significantly overexpressed in GC tissues, and that is elevated levels correlate with poorer patient prognosis. These findings underscore that GLT8D1 affects GC cell proliferation and apoptosis by modulating the expression level of PTPN6, a key regulator of the JAK2/STAT3 signaling pathway. This pathway is an integral part to the cellular processes that GLT8D1 is implicated in controlling. Despite the advancements made in this study, there are still obvious limitations that need to be further explored. Firstly, the precise molecular mechanisms underlying the negative correlation between GLT8D1 and PTPN6 remain to be fully elucidated. Understanding this relationship could provide critical insights for the development of targeted therapies. Secondly, the identification of GLT8D1-specific inhibitors that could be utilized in GC treatment has not yet been achieved. The discovery of such inhibitors may have significant implications for GC therapeutics. Addressing these limitations will be paramount in our future research endeavors.

## Conclusions

Our findings indicate that GLT8D1 is significantly upregulated in GC tissues and is associated with poor prognosis. We have discovered a novel regulatory mechanism by which GLT8D1 modulates GC cells behavior, specifically their proliferation and apoptosis. This modulation occurs by affecting the expression levels of PTPN6, a key node in the regulation of the JAK2/STAT3 signaling pathway, which is essential for various cellular processes.

## Supplementary Material

Supplementary tables.

## Figures and Tables

**Table 1:**
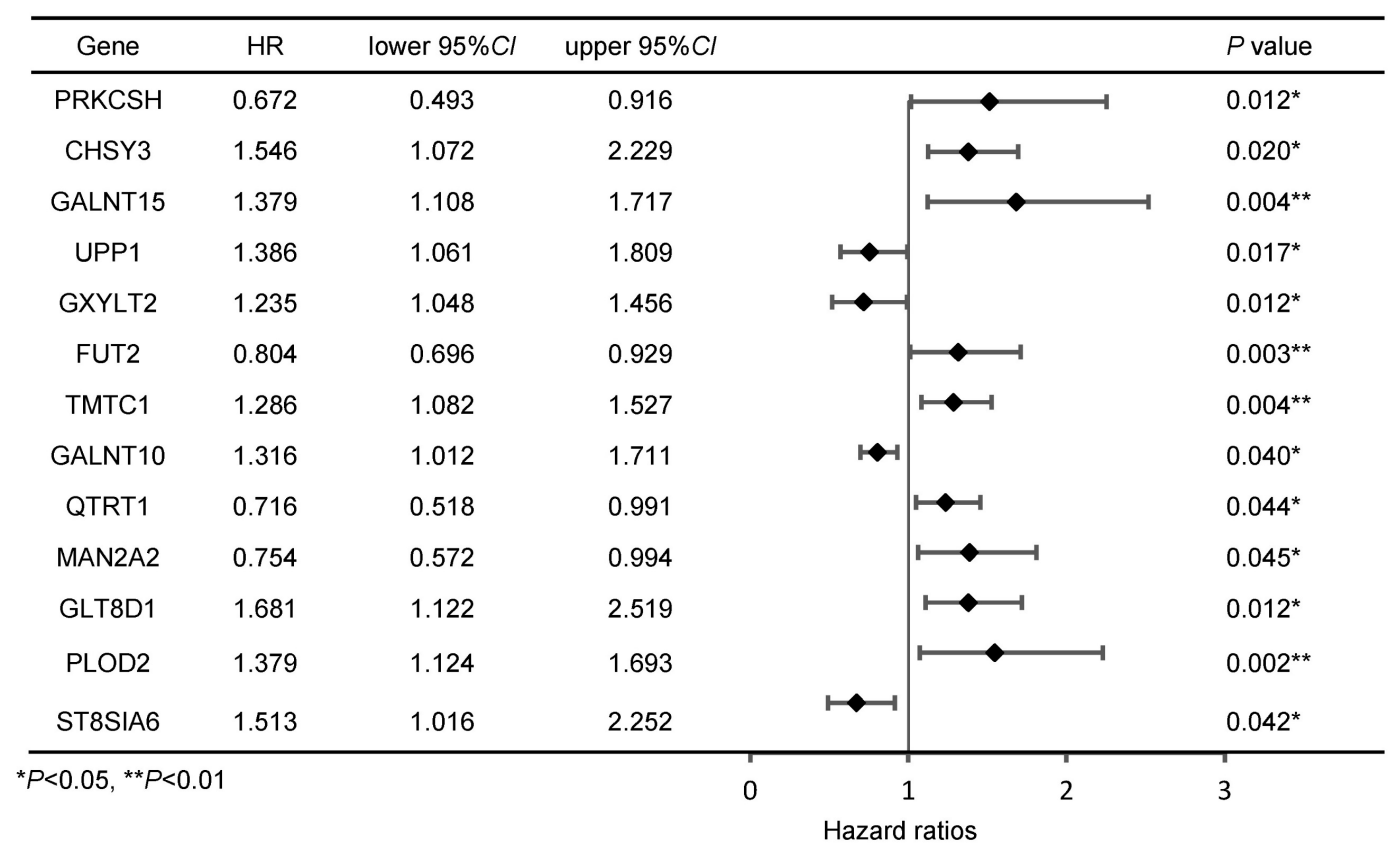
Cox regression analysis of genes associated with prognosis in GC patients

**Table 2:** Correlation between GLT8D1 expression and clinical pathological characteristics in 80 GC patients

Characteristic		All cases	GLT8D1 expression		Chi-square	*P* value
			low	high		
All cases		80	28	52		
Age	≤60	36	12	24	0.080	0.777
	>60	44	16	28		
Gender	male	57	17	40	2.334	0.127
	female	23	11	12		
Grade	G1/2	22	7	15	0.135	0.713
	G3	58	21	37		
T stage	T1/2	28	15	13	6.531	0.011^*^
	T3/4	52	13	39		
N stage	N0	31	17	14	8.756	0.003^**^
	N1-3	49	11	38		
TNM stage	I/II	38	20	18	9.891	0.002^**^
	III/IV	42	8	34		

^*^P<0.05, ^**^P<0.01

## Abbreviations

GC: gastric cancer
GLT8D1: glycosyltransferase 8 domain containing 1
RNA-seq: RNA sequencing
WGCNA: Weighted Gene Co-expression Network Analysis
PTPN6: protein tyrosine phosphatase non-receptor type 6
TCGA: The Cancer Genome Atlas
GO: Gene Ontology
FBS: fetal bovine serum
PCR: Polymerase Chain Reaction
shRNA: short hairpin RNA
qRT-PCR: quantitative reverse transcription-polymerase chain reaction
cDNA: complementary DNA
H&E: hematoxylin and eosin
IHC: Immunohistochemistry
CCK-8: Cell Counting Kit-8
EdU: 5-Ethynyl-2'-deoxyuridine
PI: propidium iodide
DEGs: differentially expressed genes
KEGG: Kyoto Encyclopedia of Genes and Genomes
OS: overall survival
PPI: protein-protein interaction
PBST: Phosphate-Buffered Saline with Tween-20
TBST: Tris-Buffered Saline with Tween
