# Supplementary material for: Overexpression of Glycosyltransferase 8 Domain Containing 1 Promotes Gastric Cancer Proliferation and Inhibits Apoptosis via Mediating PTPN6/JAK2/STAT3 Signaling Axis
Source: Int J Med Sci. 2024 Nov 11;21(15):2943–58. doi: 10.7150/ijms.102719 (PMC11610327; doi:10.7150/ijms.102719)
Supplement: Supplementary file 1 — Supplementary tables. [file ijmsv21p2943s1.pdf]

Table S1

Sequences of shRNAs and primers

**shRNAs Used for Transduction**	
**shRNAs**	**Sequence**
sh1#	CCTCAGCTTGAGCAGTTTGTTCTCGAGAACAAACTGCTCAAGCTGAGG
sh2#	GCCATATACATGGATGATGATCTCGAGATCATCATCCATGTATATGGC
sh3#	CGGAATGGAAACGACAGAATACTCGAGTATTCTGTCGTTTCCATTCCG
**Primers Used for Quantitative Real-Time PCR**	
**Gene**	**Primer sequence**
GAPDH	
Forward	GGAGCGAGATCCCTCCAAAAT
Reverse	GGCTGTTGTCATACTTCTCATGG
GLT8D1	
Forward	TTTGCACCATAACTTCCTCA
Reverse	TCTCCTCTTGTCTCCCATCTA
Bcl-2	
Forward	GGTGGGGTCATGTGTGTGG
Reverse	CGGTTCAGGTACTCAGTCATCC
Bax	
Forward	CCCGAGAGGTCTTTTTCCGAG
Reverse	CCAGCCCATGATGGTTCTGAT
P21	
Forward	TGTCCGTCAGAACCCATGC
Reverse	AAAGTCGAAGTTCCATCGCTC
c-Myc	
Forward	GGCTCCTGGCAAAAGGTCA
Reverse	CTGCGTAGTTGTGCTGATGT
PTPN6	
Forward	GGAGAAGTTTGCGACTCTGAC
Reverse	GCGGGTACTTGAGGTGGATG

Table S2

Antibodies	Source	Cat No.	Dilution	Molecular weight(kD)
Anti-Mouse GAPDH	Proteintech	60004-1-Ig	1:5000	36
Anti- Rabbit GLT8D1(WB)	CUSABIO	CSB-PA715010LA01HU	1:1000	42
Anti- Rabbit GLT8D1(IHC/IF)	Invitrogen	PA5-32140	1:100	
Anti- Rabbit Bcl-2	Abcam	ab182858	1:2000	26
Anti- Rabbit Bax	Abcam	ab32503	1:2000	21
Anti- Rabbit Cleaved CASP3	CST	9664S	1:1000	17
Anti- Rabbit P21	Proteintech	10355-1-AP	1:1000	18
Anti- Rabbit c-Myc	Proteintech	10828-1-AP	1:2000	50
Anti- Rabbit Ki-67 (IHC)	Proteintech	27309-1-AP	1:2000	
Anti-Mouse PTPN6 (WB)	Santa	sc-7289	1:500	68
Anti-Mouse PTPN6 (IF)	Santa	sc-7289	1:50	
Anti- Rabbit STAT3	CST	9139S	1:1000	88
Anti- Rabbit p-STAT3	Abcam	ab76315	1:1000	88
Anti- Rabbit JAK2	CST	3230S	1:1000	125
Anti- Rabbit p-JAK2	CST	3771S	1:1000	125
Secondary goat anti-rabbit antibody	Beyotime	A0208	1:2000	
Secondary goat anti-mouse antibody	Beyotime	A0216	1:2000	
